# The British Medical Association in London

**Published:** 1895-07-27

**Authors:** 


### The Hospital

An Institutional Journal of

The Medical Sciences and Hospital Administration, with

Vol. XVIII.—No. 461.] AN EXTRA NURSING SUPPLEMENT. [July 27, 1895.

### The British Medical Association in London.

It is the enduring distinction of the medicine of the nineteenth century that it has adopted absolute science as its master and guide, and has made man in his entirety—the individual and the race—the object of its study. "The proper study of mankind is man": so philosophy, ancient and modern, is always telling us. Men of science devote their lives to every conceivable subject in heaven, earth, and the sea. But when we place every subject of scientific study side by side for comparison, we see, without a moment's doubt or hesitation, that man, the reasoner; man, the sufferer; man, the individual; man, the race, is still the crown and summit of all that science can know or will ever strive to know. The most distinguished students of reasoning, working, and suffering man which the world has ever known will meet together in congress in London, under the auspices of the British Medical Association, during the closing days of the present month and the opening days of the next.

The difficulties of the subjects which the Congress meets to discuss, and their present remoteness from common knowledge, deprive them to a great extent of interest for the average man. Not being of absorbing interest to the man in the street, they run the risk of being passed over with very inadequate notice by those whose business it is to furnish the public with information and instruction day by day, that is, by the non-professional newspapers. If such should prove to be the case the public and the world will be serious losers.

It is readily appreciated by all men that improvements in machinery and in weapons of war are the surest indications and guarantees of material progress and safety. The great aim of modern medicine is the improvement and sublimation of that machine of machines, the human body, with its brain and mind. It cannot be denied that the scientific engineers of the human body and mind are men of rare powers and rare honesty. It cannot be affirmed that the subjects which they will discuss with their best powers and their severest honesty are of no importance to mankind. If then those ablest men in the world, discussing subjects of first-rate importance to mankind, excite no interest in the man in the street or in the writers of his newspapers, must it not be that both the man in the street and the writers of his newspapers are ignorant of things in which they ought to be instructed, and indifferent to subjects in which they ought to take the keenest interest that man can feel? We reason thus not because we desire that medicine and its doings may fill the public eye during the week of its Congress, but that the public may make an effort, for its own advantage, to understand those great things which modern medicine is doing and is labouring to do, and may give its earnest endeavours a heartfelt sympathy and help which will be felt as an inspiring and enduring force to the remotest bounds of medical and human science in every part of the world.

The brain is the chiefest of all the instruments of human progress. Two of the sectional departments of the Congress—the sections of psychology and physiology—will bring to a focus all the knowledge which experiment and every variety of research have accumulated on this great subject up to the present time. Those sections will discuss not only the profoundly interesting records of the past, but the absorbing possibilities of the future. Similarly those vast and varied areas of minute life which are classed together under the title of "Bacteriology" will reveal to a wondering world the marvels and miracles which are associated with the infinitely little. Anatomy and histology, arid, perhaps, in themselves, become instinct with living and breathing interest, when once they are studied as the physical bases of the pains and pleasures, the failures and triumphs, of every form of human thought and endeavour. Ophthalmology, which will be studied in a separate section, makes its appeal to the artist and the lover of scientific wonders as well as to the practical person who sees nothing in the eye but a sensitive instrument of perception. The great subject of general medicine, and that of general surgery, will each be dealt with in a distinct section, as will also the subjects of obstetrics, public medicine, pathology, diseases of children, otology, laryngology, dermatology, pharmacology, and therapeutics. The subject of ethics, for the first time, we believe, in the history of the British Medical Association, will be given over to a section for its more precise and scientific study. For the present, no doubt, attention will be limited to what are called "medical ethics." It is quite certain, however, that in no long time physiology and medicine will insist upon making authoritative pronouncement in other departments of ethics, in criminal and political ethics, perhaps, especially; but sooner or later in the whole field of this great and vitally important subject.

In short, a battalion of the "noble army of science" will turn out for a series of London field-days during the last week of July and the first week of August. A unique opportunity will be given to those leaders of everyday thought, the general newspapers, and to the intelligent public, to see and hear and critically measure up some of the most scientifically potent persons in the world. It is important for two reasons that the non-scientific should see and judge the scientific. The scientific may thus learn how and wherein they come short in the light they reveal and the services they render to mankind; and the non-scientific may perhaps be brought to recognise how high a standard of truthfulness, simplicity, and transparent candour is set up before them as a noble object lesson by the workers in science. Above all, the citizens of the metropolis and the strangers within our gates will have an opportunity of seeing, as in a bird's-eye view, all the great and far-reaching things of a practical order which modern medicine and physiology are strenuously and heroically striving to do for the world. As individuals many medical men may be of small account; in general politics we may be "a feeble folk"; but as scientific pioneers in the world's struggle towards higher, happier, and more victorious things who is there in modern science whose record is nobler than ours? Can any science say truthfully of its workers that they have done more to ameliorate human ills and to promote the happiness of the human family than scientific medicine and surgery have done? If this be indeed so, let the great world for once turn aside from its less important to its more important activities and give a grateful recognition to those who, though ordinarily out of its sight, yet unceasingly labour for its enlightenment, its happiness, its health, and its life.

